# EDEM1 Regulates Amyloid Precursor Protein (APP) Metabolism and Amyloid-β Production

**DOI:** 10.3390/ijms23010117

**Published:** 2021-12-23

**Authors:** Jowita Nowakowska-Gołacka, Justyna Czapiewska, Hanna Sominka, Natalia Sowa-Rogozińska, Monika Słomińska-Wojewódzka

**Affiliations:** Department of Medical Biology and Genetics, Faculty of Biology, University of Gdańsk, Wita Stwosza 59, 80-308 Gdansk, Poland; jowita.nowakowska-golacka@ug.edu.pl (J.N.-G.); justyna-czapiewska@wp.pl (J.C.); hanna.sominka@phdstud.ug.edu.pl (H.S.); natalia.sowa@phdstud.ug.edu.pl (N.S.-R.)

**Keywords:** endoplasmic reticulum degradation-enhancing α-mannosidase-like 1 protein (EDEM1), amyloid-β precursor protein (APP), endoplasmic reticulum-associated degradation (ERAD), amyloid-β (Aβ), endoplasmic reticulum (ER), protein degradation, protein quality control

## Abstract

Endoplasmic reticulum (ER) degradation-enhancing α-mannosidase-like protein 1 (EDEM1) is a quality control factor directly involved in the endoplasmic reticulum-associated degradation (ERAD) process. It recognizes terminally misfolded proteins and directs them to retrotranslocation which is followed by proteasomal degradation in the cytosol. The amyloid-β precursor protein (APP) is synthesized and N-glycosylated in the ER and transported to the Golgi for maturation before being delivered to the cell surface. The amyloidogenic cleavage pathway of APP leads to production of amyloid-β (Aβ), deposited in the brains of Alzheimer’s disease (AD) patients. Here, using biochemical methods applied to human embryonic kidney, HEK293, and SH-SY5Y neuroblastoma cells, we show that EDEM1 is an important regulatory factor involved in APP metabolism. We find that APP cellular levels are significantly reduced after EDEM1 overproduction and are increased in cells with downregulated EDEM1. We also report on EDEM1-dependent transport of APP from the ER to the cytosol that leads to proteasomal degradation of APP. EDEM1 directly interacts with APP. Furthermore, overproduction of EDEM1 results in decreased Aβ_40_ and Aβ_42_ secretion. These findings indicate that EDEM1 is a novel regulator of APP metabolism through ERAD.

## 1. Introduction

Alzheimer’s disease (AD) is irreversible, progressive, and the most common brain disorder that results in dementia, mainly in the elderly. A very characteristic and dominant neuropathological hallmark of this disease is associated with the accumulation of the amyloid-β peptide (Aβ) in the senile plaques [1,2,3]. These plaques are deposited extracellularly, in the grey matter of the brain; however, an aggregation-prone Aβ is produced inside the cells [4,5,6,7].

Aβ is formed by sequential proteolytic cleavage of a large, type 1 transmembrane glycoprotein, the amyloid-β precursor protein (APP), that is ubiquitously expressed in the neuronal and non-neuronal cells (for a review see for example references [8,9]). During its translation, APP is incorporated into the endoplasmic reticulum (ER) membrane, thus entering the constitutive secretory pathway. Along this pathway, APP maturates by being subjected to extensive post-translational modifications that affect its sorting and trafficking (for a review see reference [10]). The immature, N-glycosylated APP is mostly located in the ER. The mature APP, which is additionally O-glycosylated, as well as subjected to other modifications (e.g., tyrosyl-sulfation, palmitoylation, and phosphorylation), is mainly detected in the trans-Golgi network (TGN) or at the plasma membrane. However, different forms of APP are much more diverse, as there are about ten variants of this protein arising from alternative splicing of exons 7 and 8. The length of these isoforms varies from 639 to 770 amino acids. The three major isoforms: APP_695_, APP_751_, and APP_770_ are the most significant. Both the full-length APP_751_ and APP_770_ have a Kunitz-type serine protease inhibitors (KPI) domain, while APP_770_ also contains a 19-amino acid OX-2 domain which follows the KPI domain (for a review see references [10,11]). All of the main isoforms of APP can generate Aβ by a metabolic pathway termed the amyloidogenic pathway. Some evidence shows that APP_695_ is preferentially involved in Aβ production [12], whereas other evidence demonstrates that APP_751_ and APP_770_ overexpression in the brain is primarily associated with the Aβ release [13]. Moreover, it is not clear whether APP isoforms trigger the amyloidogenic pathway in the same way. However, it is known that amyloid production involves cleavage by membrane-bound aspartyl proteases, the β-site APP cleaving enzyme (BACE) 1 or BACE2, and subsequently by the presenilin-γ-secretase complex [14,15,16]). Cleavage by secretases results in formation of 39–43 residue peptides, among which Aβ_40_, as well as Aβ_42_, become components of the senile plaques. However, it was proven that Aβ_42_ is more hydrophobic and amyloidogenic than other forms, being preferentially deposited in the AD brains [17,18]. It cannot be excluded that Aβ can be generated in any of the organelles along the secretory pathway. The γ-secretase is predominantly localized in unique subcompartments of the ER or in the ER to Golgi trafficking intermediate vesicles [19], while Aβ_42_ is present in the ER and can be exported from microsomes [20]. However, it is suggested that production of Aβ takes place mainly in the Golgi or trans-Golgi network (TGN) [21,22,23], and that endocytosed APP, which is recycled from endosomes to the TGN, is important in this process [6]. Significantly, the amyloidogenic pathway is not predominant during the APP proteolytic processing. It is assumed that more than 90% of the processed APP undergoes a non-amyloidogenic pathway [11]. This processing involves sequential cleavage by α- and γ-secretases. The α-secretase acts mainly at the plasma membrane and results in the production of an N-terminal secreted APP (sAPPα) that has been shown to be mostly neurotropic with an ability to counteract the pathogenic effects of Aβ [24,25,26].

The early stages of APP maturation that occur in the ER are crucial for the intracellular metabolism of this protein and subsequent Aβ production. N-glycosylation is solely a part of a much more complex process that is related to proper folding of APP in the ER. This organelle possesses a large number of specifically acting enzymes, molecular chaperones and quality control factors that are responsible for proper folding of newly synthesized glycoproteins (for a review see references [27,28]). The ER glycoprotein folding quality control (ERQC) determines the maturation of APP. It has been demonstrated that APP interacts with the Hsp70 chaperone family protein, BiP/GRP78 [29] and calreticulin [30,31], a specific ER lectin chaperone. Both BiP and calreticulin directly facilitate proper folding of glycoproteins in the ER. Moreover, it was shown that the interaction of APP with BiP reduces Aβ_40_ and Aβ_42_ secretion [29]. ERQC ensures that only properly folded proteins are secreted into their intended compartments in the cell, whereas terminally misfolded glycoproteins are directed for degradation in a process termed ER-associated protein degradation (ERAD) (for a review see references [32,33,34,35]). Degradation takes place in the cytosol and is carried out by the 26S proteasome complex. Therefore, protein substrates in the ERAD process must be first recognized in the ER, targeted to a specific ER membrane translocon, and transported into the cytosol for degradation. It has been reported that APP retrotranslocates from the ER to the cytosol and that it co-immunoprecipitates with Derlin-1 [36], a member of the ERAD retrotranslocation machinery [37,38]. It was also proven that APP becomes ubiquitinated and degraded by the proteasome. HRD1, an ER ubiquitin-ligase E3, was shown to be involved in APP ubiquitination and degradation, which subsequently resulted in decreased Aβ production [39,40]. Moreover, it was demonstrated that FBL2, a component of the E3 ubiquitin ligase complex, promotes APP ubiquitination and its further proteasomal degradation, resulting in decreased secretion of Aβ_40_ and Aβ_42_ [41].

It seems very likely that APP must be specifically recognized in the ER before its transport to the cytosol for proteasomal degradation. The ER degradation-enhancing α-mannosidase-like protein 1 (EDEM1) has been demonstrated to recognize and extract terminally misfolded proteins from productive folding cycles, before targeting them for degradation by ERAD [42,43,44]. Removal of the α1,2 mannose residues from the exposed N-glycans is the signal for ERAD indicating misfolded glycoproteins in mammalian cells (for a review see references [33,45,46]). Interestingly, EDEM1 has a mannosidase activity, as demonstrated in vivo [47,48,49] and in vitro [50]. However, interactions of EDEM1 with ERAD substrates, as well as with ERAD regulators, are complex and multifaceted [51,52,53,54,55,56,57,58]. In our previous studies, we demonstrated that EDEM1 can recognize an untypical ERAD substrate, the ricin-A chain, and promotes its retrotranslocation from the ER to the cytosol [59,60,61]. Importantly, appropriate substrate protein structure, as well as its high hydrophobicity, are relevant for interactions with EDEM1 [62,63].

In this study, we investigated the effects of EDEM1 on APP metabolism and Aβ secretion. We report that EDEM1 regulates cellular levels of APP by promoting transport of the immature form of APP from the ER to the cytosol, where APP is degraded by the proteasome. This results in decreased generation of Aβ_40_ and Aβ_42_. Together, our findings provide direct evidence that the APP metabolism is dependent on EDEM1, a key regulator of the ERAD process.

## 2. Results

### 2.1. Intracellular Level of APP Is Significantly Reduced in the HEK293 Cells Overexpressing EDEM1

**Figure 1:**
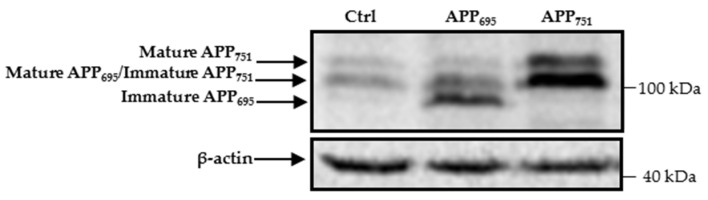
HEK293 cells express different APP isoforms. Western blotting of APP and actin in cell lysates of HEK293 transfected with control, APP_695,_ or APP_751_ cDNAs. The different APP isoforms are indicated by arrows on the left side of the membrane. Immature and mature forms of APP_770_ were not analyzed in this work and therefore location of these isoforms is not indicated. Molecular mass markers are shown on the right.

Human embryonic kidney 293 (HEK293) cells produce endogenous forms of APP_695_, APP_751,_ and APP_770_, as was previously described in [12]. The immature form of APP_695_ is poorly detected by Western blotting, whereas it is clearly visible in lysates of cells with overproduction of this protein (Figure 1). The endogenous immature form of APP_751_, together with mature APP_695_ and the mature form of APP_751_ are expressed at relatively high levels (Figure 1). Nevertheless, these levels are significantly higher in the APP_751_-transfected cells than in the cells transfected with control cDNA (Figure 1).

**Figure 2:**
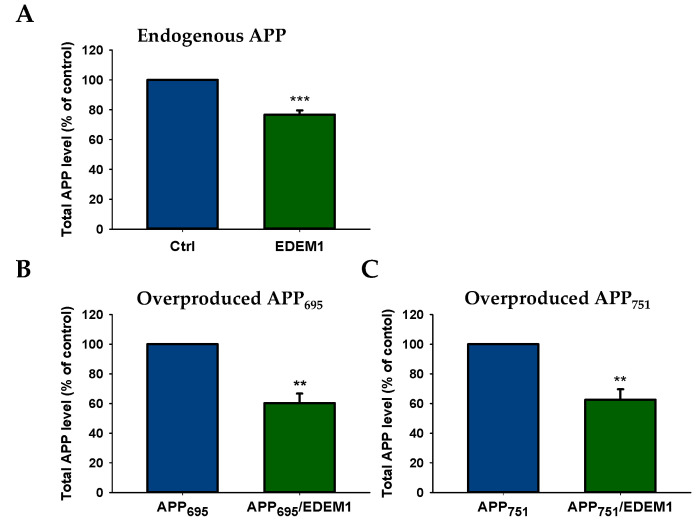
Total amount of APP is decreased in EDEM1-transfected HEK293 cells. ELISA analysis of (**A**) endogenously expressed APP, (**B**) overproduced APP_695_, or (**C**) overproduced APP_751_ in lysates from cells with or without EDEM1 cotransfection. The values are expressed as mean ± SD, *n* = 3, ** *p* ˂ 0.01, *** *p* ˂ 0.001, Student’s *t* test. EDEM1 overexpression and equal β-actin levels were assessed by Western blotting, as indicated in Figure 3.

To verify whether the intracellular levels of both the endogenous and overproduced forms of APP could be regulated by high EDEM1 production, we examined the levels of APP_695_ and APP_751_ in HEK293 cells by ELISA. For this purpose, cells were transfected with EDEM1 cDNA or cotransfected with APP_695_ and EDEM1 or APP_751_ and EDEM1 expression vectors (for transfection conditions, see Section 4). The ELISA analysis of total APP in EDEM1-transfected cells shows a decrease in endogenously produced protein by approximately 25%, relative to control cells (Figure 2A), and an approximately 40% decrease in cells overproducing APP_695_ or APP_751_ (Figure 2B,C). It should be noted that in cells with overproduction of APP_695_ (Figure 2B) or APP_751_ (Figure 2C), total amount of all APP isoforms was analyzed.

**Figure 3:**
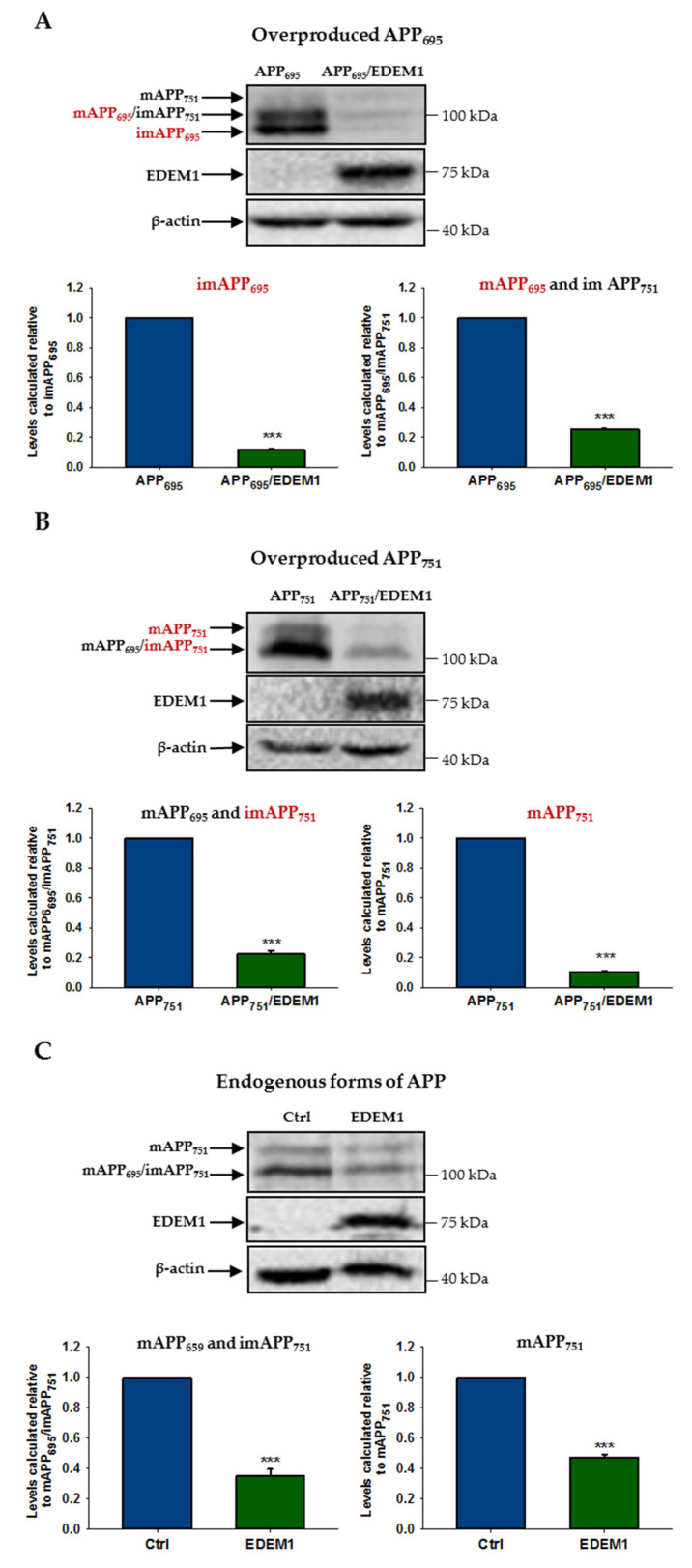
The level of various APP isoforms is reduced in HEK293 cells overexpressing EDEM1. Western blotting of APP, EDEM1 and actin in lysates of cells transfected with (**A**) APP_695,_ (**B**) APP_751,_ or (**C**) control cDNA with or without EDEM1 cotransfection (as indicated). Overexpressed, dominant forms present in (**A**,**B**) are indicated in red. Mature APP is abbreviated as mAPP, and immature APP as imAPP. Representative experiments are shown. Molecular mass markers are shown on the right side of the membranes. The levels of APP isoforms were quantified and are shown in the graphs. Values obtained for lysates of cells without EDEM1 overexpression are indicated as 1. Values representing EDEM1-transfected cells are plotted relative to 1. The values are expressed as mean ± SD, *n* ≥ 3, *** *p* ˂ 0.001, Student’s *t* test.

To analyze reduction in the level of individual forms of APP_695_ and APP_751_ by EDEM1 overproduction, we assessed the level of APP by Western blotting in lysates from EDEM1-transfected HEK293 cells or cells cotransfected with APP_695_ and EDEM1 or APP_751_ and EDEM1. As shown in Figure 3, overproduction of EDEM1 significantly reduced the amount of both the immature and mature forms of APP_695_ and APP_751_. The most spectacular effect was observed for the immature N-glycosylated form of APP_695_, where a more than eight-fold decrease in the amount of this form was detected (Figure 3A). A huge decrease was also found for the overproduced mature form of APP_751_ (Figure 3B). The reduction in endogenously expressed forms of APP_695_ and APP_751_ was significant (Figure 3C), but not as great as that for overexpressed APP. This may suggest that EDEM1 is especially involved in the APP metabolism when a large amount of this protein is accumulated in the ER. Probably, regulation of the amount of immature forms of APP_695_ and APP_751_ by EDEM1 has a direct effect on the intracellular level of mature forms. However, the EDEM1-related reduction in the level of immature and mature forms of APP_695_ and APP_751_ is slightly different (Figure 3A,B), which may suggest that their intracellular levels may still depend on other distinct regulatory proteins.

**Figure 4:**
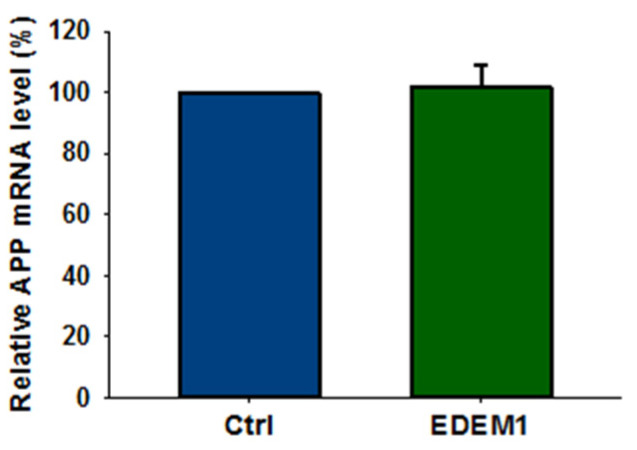
High expression of EDEM1 does not affect APP transcription in HEK293 cells. Relative APP mRNA level in HEK293 cells expressing EDEM1 was quantitatively assessed by real-time RT-PCR. Measurements were performed with the use of the Roche Universal Probe Library (UPL) assays. Expression of GAPDH mRNAs was used as reference control. Values obtained for cells without EDEM1 overexpression are indicated as 100%. Values representing EDEM1-transfected cells are plotted relative to 100%. The values are expressed as mean ± SD, *n* = 3, Student’s *t* test.

It should be noted that overproduction of EDEM1 does not affect APP gene transcription (Figure 4), and thus decrease in the amount of APP by EDEM1 overproduction is regulated at the post-transcriptional level.

### 2.2. Overproduction of EDEM1 Regulates the APP Level through Proteasomal Degradation

**Figure 5:**
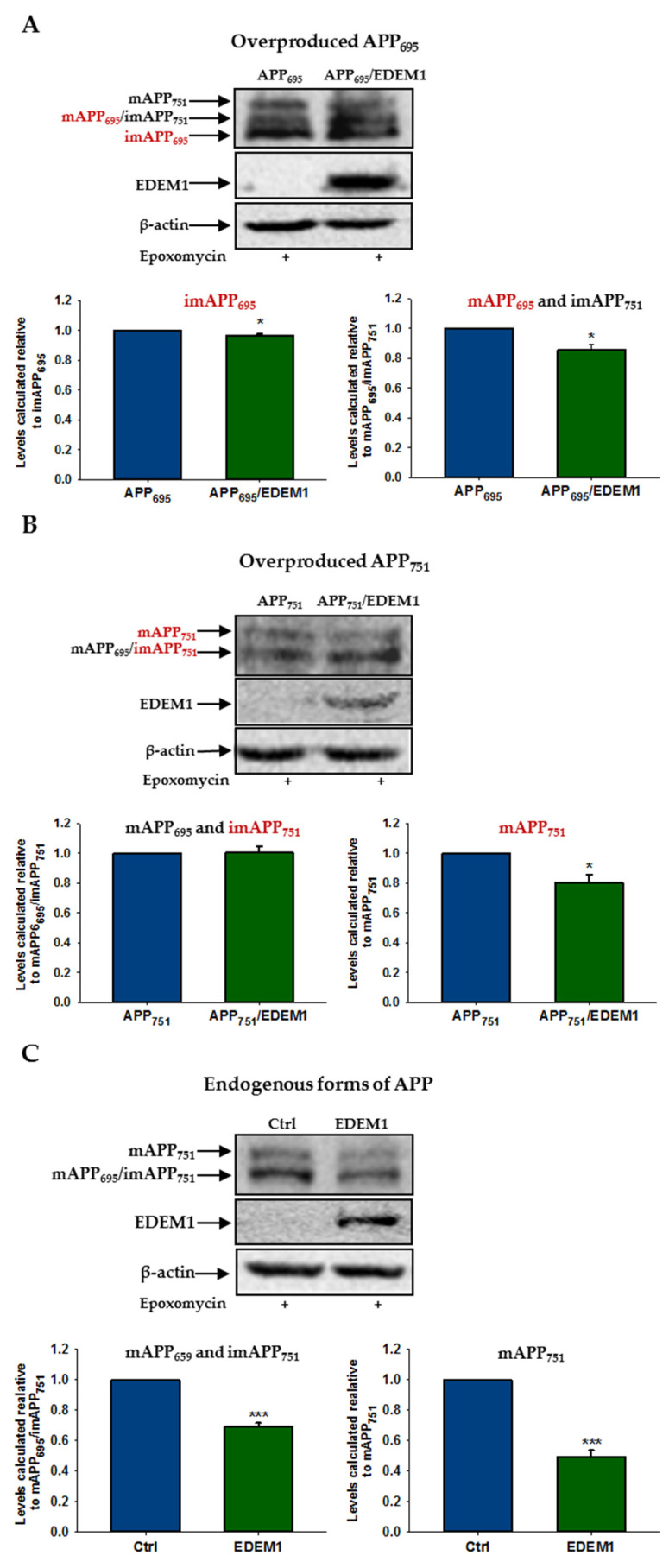
The effect of EDEM1-dependent intracellular reduction on the level of APP isoforms is significantly attenuated after proteasome inhibition. Western blotting of APP, EDEM1 and actin in lysates of cells transfected with (**A**) APP_695_, (**B**) APP_751_, or (**C**) control cDNA with or without EDEM1 cotransfection (as indicated). Cells were treated with 10 µM lactacystin or 1 µM epoxomicin for 5 h before cell lysis. Overexpressed, dominant forms present in (**A**,**B**) are indicated in red. Representative experiments are shown for cells treated with epoxomicin. Similar results were obtained for cells treated with lactacystin. The levels of APP isoforms were quantified and are shown in the graphs. Values obtained for lysates of cells without EDEM1 overexpression are indicated as 1. Values representing EDEM1-transfected cells are plotted relative to 1. The values are expressed as mean ± SD, *n* ≥ 3, * *p* ˂ 0.05, *** *p* ˂ 0.001, Student’s *t* test.

Since the 26S proteasome is largely responsible for intracellular degradation of APP [39,40,64,65,66], we studied the effect of a proteasome inhibitor on APP accumulation under EDEM1 overproduction. For this purpose, lactacystin or epoxomicin, selective and irreversible proteasome inhibitors, were applied. As shown in Figure 5, proteasome inhibition significantly abolishes the effect of EDEM1-induced intracellular reduction on the amount of immature and mature forms of APP_695_ and APP_751_. This includes both the endogenous and overproduced forms of APP. Interestingly, the intracellular levels of immature overproduced APP_695_ and APP_751_ in cells with EDEM1 overproduction which are treated with a proteasome inhibitor almost fully correspond to the amounts of these forms in the cells without EDEM1 overproduction (Figure 5A,B).

Thus, it can be concluded that the EDEM1-dependent intracellular reduction in APP level is mainly associated with the proteasomal degradation of this protein in the cytosol. This effect is particularly noticeable in the case of immature, overproduced forms of APP.

### 2.3. APP Retrotranslocation from the ER to the Cytosol Is Regulated by EDEM1 in HEK293 Cells

**Figure 6:**
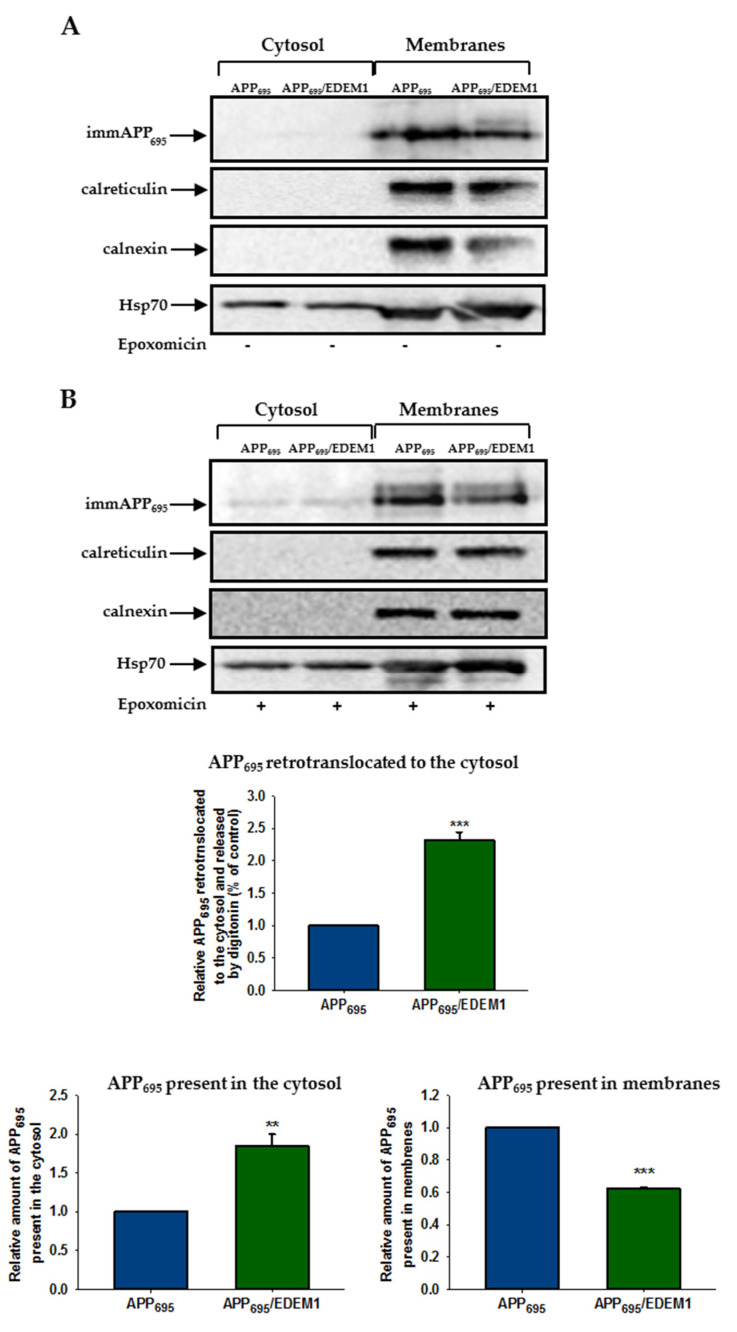
APP_695_ retrotranslocation from the ER to the cytosol is dependent on EDEM1. Retrotranslocation assay of APP_695_ in APP_695_-transfected cells or cells cotransfected with APP_695_ and EDEM1. Immature APP is abbreviated as imAPP. Cells were either (**A**) not treated with epoxomicin, or (**B**) treated with epoxomicin (1 µg/mL) for 5 h before the permeabilisation procedure was applied. Representative examples of Western blotting of APP_695_, calnexin, calreticulin, and Hsp70 present in the cytosolic and membrane fractions are shown. The graphs show the relative amounts of APP_695_ retrotranslocated to the cytosol, present in the cytosol, or present in the membranes in cells treated with epoxomicin. Values obtained for lysates of cells without EDEM1 overexpression are indicated as 1. Values representing EDEM1-transfected cells are plotted relative to 1. The values are expressed as mean ± SD, *n* = 3, ** *p* ˂ 0.01,*** *p* ˂ 0.001, Student’s *t* test.

It has previously been demonstrated that APP is retrotranslocated from the ER to the cytosol in CHO_APP751_ cells [36]. To assess whether EDEM1 overproduction affects APP_695_ retrotranslocation out of the ER in HEK293 cells, we applied a permeablisation assay that we have previously used to study the ricin toxin A-chain retrotranslocation from the ER to the cytosol [59,60,62]. The HEK293 cells overproducing APP_695_ or cells cotransfected with APP_695_ and EDEM1 were semipermeablised with a mild detergent (digitonin) to obtain separate cytosolic and membrane fractions. It was shown before that treatment of cells with the proteasome inhibitor epoxomicin enables cytosolic accumulation of the immature form of APP_695_, whereas without proteasome inhibition this APP form was not detectable [36]. These findings were confirmed in our studies (Figure 6A). For cells treated with epoxomicin, our results indicate an approximately two-fold increase in APP_695_ retrotranslocation from the ER to the cytosol in EDEM1 overproducing cells when compared to control cells (Figure 6B). It should be noted that a higher amount of the immature form of APP_695_ was observed in the cytosolic fraction and there was a concomitantly reduced level of APP_695_ in the membranes of EDEM1 overexpressing cells when compared to the corresponding cytosolic and membrane fractions of cells without EDEM1 overproduction (Figure 6B). Importantly, accumulation of APP_695_ in the cytosol was not due to an uncontrolled leakage of APP out of the ER. To verify this, we examined localization of calreticulin, a soluble ER protein, and calnexin, an ER membrane protein, in cells subjected to permeabilisation. As shown in Figure 6A,B, these proteins were not released from the ER into the cytosol, indicating that the ER membranes remained intact. Thus, it can be concluded that high production of EDEM1 promotes retrotranslocation of the immature form of APP_695_ to the cytosol.

### 2.4. Downregulation of EDEM1 Causes a Significant Elevation in APP Amounts in the HEK293 and SH-SY5Y Cells

**Figure 7:**
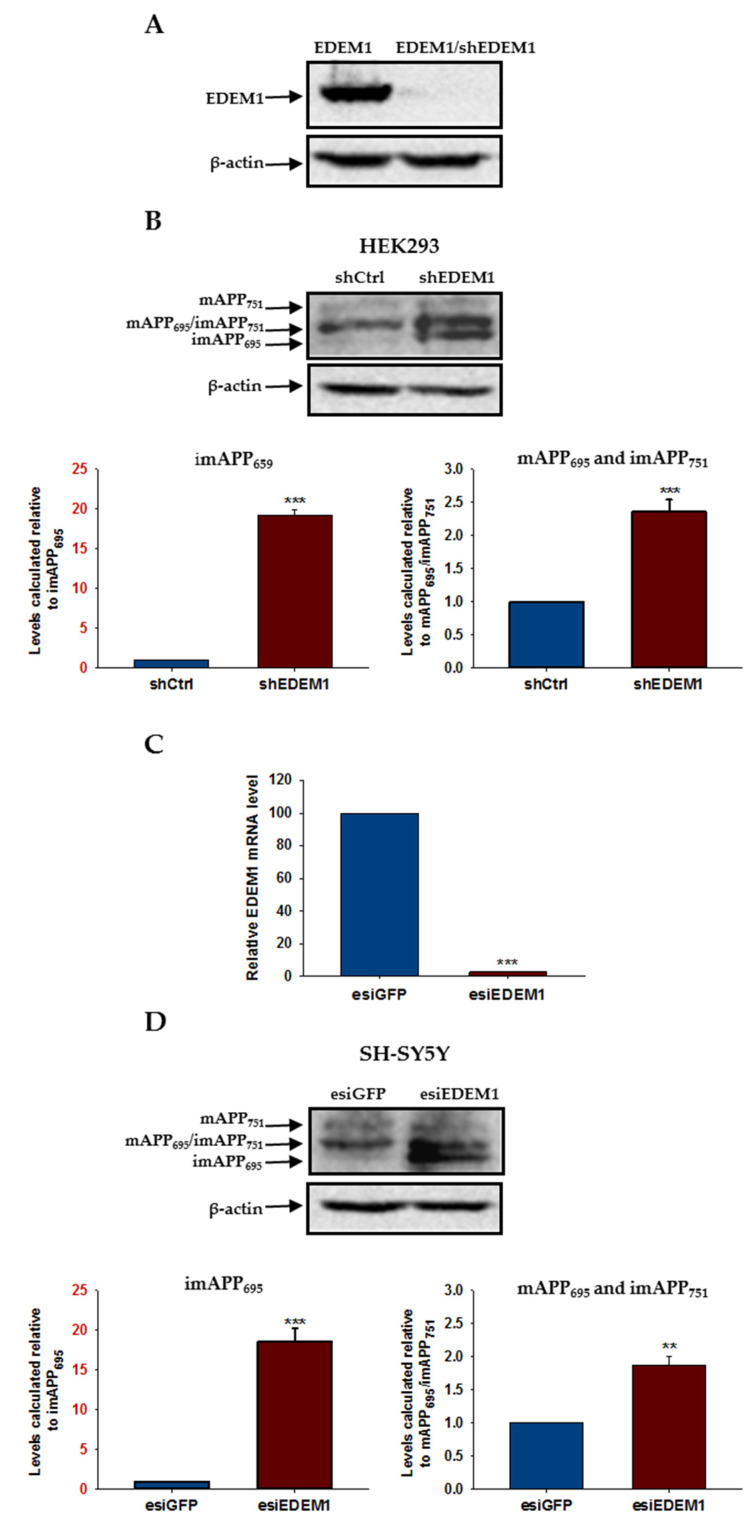
APP isoform levels are increased in HEK293 and SH-SY5Y cells with downregulated EDEM1. EDEM1 mRNA levels were analyzed by qRT-PCR or Western-blotting. (**A**) Western blotting of EDEM1 and actin in HEK293 cells transfected with EDEM1 or cotransfected with EDEM1 and shEDEM1 construct. Representative experiment is shown. (**B**) Western blotting of APP and actin in lysates of HEK293 cells with or without downregulated EDEM1 (as indicated). Representative experiment is shown for cells transfected with shEDEM1 or control shRNA (shCtrl). Mature APP is abbreviated as mAPP, and immature APP as imAPP. The level of APP isoforms was quantified and is shown in the graphs. Values obtained for lysates of cells without EDEM1 downregulation are indicated as 1. Values representing cells with reduced levels of EDEM1 are plotted relative to 1. Note that the scale of the Y axis for both graphs is not the same. The Y axis scale for imAPP_695_ is highlighted in red. The values are expressed as mean ± SD, *n* = 3, ** *p* ˂ 0.01, *** *p* ˂ 0.001, Student’s *t* test. (**C**) Relative EDEM1 mRNA level in SH-SY5Y cells expressing esiEDEM1 assessed quantitatively by real-time RT-PCR. Measurements were performed with the use of the Roche Universal Probe Library (UPL) assays. Expression of GAPDH mRNAs was used as reference control. Values obtained for cells without EDEM1 downregulation are indicated as 100%. Values representing esiEDEM1-transfected cells are plotted relative to 100%. The values are expressed as mean ± SD, *n* = 3, Student’s *t* test. (**D**) the same as in (**B**) but for SH-SY5Y cells transfected with esiEDEM1 or control esiRNA (esiGFP).

To analyze the relationship between EDEM1 and APP in more detail, we first examined the levels of endogenous forms of APP in HEK293 cells with reduced intracellular amounts of EDEM1. For this purpose, cells were treated with a heterogeneous mixture of siRNA, esiRNA specific for EDEM1 (esiEDEM1), or a control unspecific esiRNA (silencing GFP, esiGFP). Alternatively, cells were transfected with short hairpin RNA, shRNA against EDEM1 (shEDEM1) [59]. Our previous experiments showed that both esiEDEM1 and shEDEM1 effectively downregulate the expression of EDEM1 [59,60]. Additionally, in each experiment, EDEM1 mRNA levels were quantified by qRT-PCR and were estimated relative to GAPDH mRNA, or protein levels were analyzed by Western blotting (Figure 7A). All analyzed data come from experiments in which highly efficient downregulation (90–95%) of EDEM1 was achieved. A reduced level of EDEM1 causes a significant elevation in intracellular levels of APP, both in the immature and mature forms (Figure 7B). Interestingly, the level of the endogenous immature form of APP_695_ increased approximately 20-fold in cells with downregulated EDEM1, reflecting the levels obtained in cells with overproduction of APP_695_ (compare with Figure 1).

To verify the generality of APP regulation by the EDEM1 chaperone protein, we downregulated EDEM1 in SH-SY5Y neuroblastoma cells. Endogenous APP has been shown to be expressed at relatively high levels in these cells [12]. For EDEM1 level reduction, cells were treated with esiEDEM1 or control esiGFP. We estimated that, as in HEK293 cells, a concentration of 40 nM esiEDEM1 caused an approximately 95% reduction in EDEM1 mRNA levels, as quantified by qRT-PCR and analyzed relatively to GAPDH mRNA (Figure 7C). Intracellular levels of both the immature and mature forms of APP were significantly increased in SH-SY5Y cells after EDEM1 downregulation (Figure 7D). Similar to HEK293 cells, an approximately 20-fold elevation in the amount of the immature form of APP_695_ was observed. These observations suggest that EDEM1 is a significant regulator of intracellular APP protein levels.

### 2.5. EDEM1 Colocalizes and Interacts with APP

**Figure 8:**
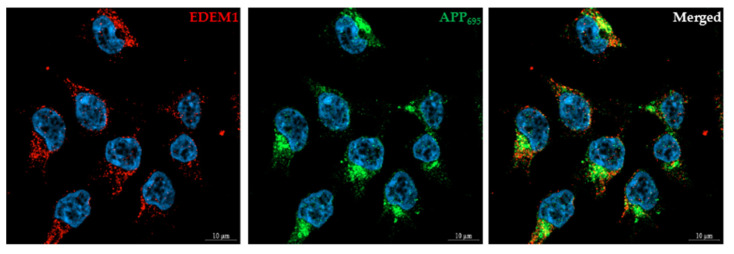
EDEM1 colocalizes with APP in HEK293 cells. Cells were transfected with APP_695_ and EDEM1, and were then fixed and stained as indicated. DAPI was used to stain the nuclei. Bars, 10 µm. Control staining without anti-APP or anti-EDEM1 antibodies confirmed specific recognition by secondary antibodies.

We first used confocal immunofluorescence imaging to examine the colocalization of EDEM1 and APP. Immunostaining of HEK293 cells cotransfected with APP_695_ and EDEM1 showed colocalization of both proteins (Figure 8), with the degree of EDEM1 colocalization with APP_695_ equal to 0.87 (Mander’s overlap coefficient, mean ± SD, *n* = 5, *** *p* ˂ 0.001). As was previously reported in [59], EDEM1 is localized in the ER. This protein was found to colocalize with the ER protein disulfide isomerase (PDI) (Supplementary Materials Figure S1), with the degree of EDEM1 colocalization with PDI equal to 0.7 (*n* = 3, *** *p* ˂ 0.001). It can be concluded that most of the EDEM1 that is localized in the ER colocalizes with APP_695_; however, the degree of APP_695_ colocalization with EDEM1 is decreased by 33% (*n* = 5, *** *p* ˂ 0.001) in comparison to EDEM1 colocalization with APP_695_. This is due to the fact that intacellular APP_695_ does not entirely localize in the ER.

**Figure 9:**
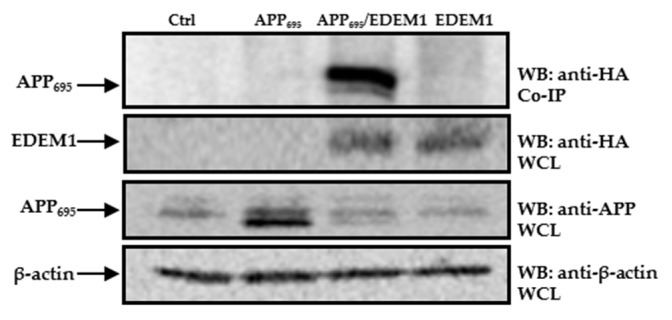
APP_695_ interacts with EDEM1. Co-immunoprecipitation of APP from lysates of cells transfected with control vector, EDEM1, APP_695,_ or cells cotransfected with APP_695_ and EDEM1. The beads were coated with anti-HA antibodies because the EDEM1-HA construct was used for transfection. Representative example is shown. Whole cell lysates (WCL) were analyzed with anti-HA, anti-APP, or anti β-actin antibodies.

To further investigate possible interactions between EDEM1 and APP_695_, and to confirm that EDEM1 is directly involved in APP transport to the cytosol, we applied a co-immunoprecipitation assay. A band corresponding to APP was detected in precipitates from cells cotransfected with APP_695_ and EDEM1, but not in precipitates from cells transfected only with APP_695_, EDEM1, or a control cDNA (Figure 9). This indicates the specificity of the observed interactions between APP and EDEM1. 

### 2.6. Intracellular Level of EDEM1 Influences Aβ Secretion

We next investigated the effect of EDEM1 overexpression on Aβ_40_ and Aβ_42_ secretion in HEK293 cells cotransfected with APP_Swe/Ind_ and EDEM1 or APP_deltaCT_ and EDEM1. The Swedish mutation of the APP protein (APP_Swe_) is known to enhance the abnormal cleavage of cellular APP by β-secretase, resulting in an increase in the total Aβ production and secretion, specifically of the Aβ_40_ and Aβ_42_ forms [67,68]. On the other hand, the APP Indiana mutation (APP_Ind_) increases the Aβ_42_/Aβ_40_ ratio in conditioned media [69,70]. Finally, in cells transfected with an APP construct with a deletion of the C-terminal intracellular domain of APP (APP_deltaCT_) [71], the detected Aβ is entirely derived from the secretory pathway. Since the intracellular domain of APP is necessary for proper endocytosis of APP, production of Aβ that comes from the endocytic pathway is blocked in cells transfected with APP_deltaCT_ [72,73].

**Figure 10:**
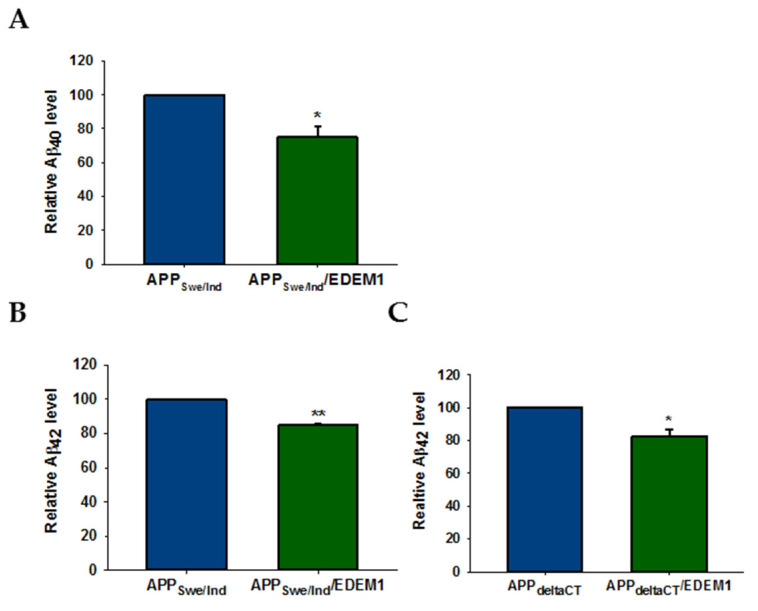
Amount of Aβ_40_ and Aβ_42_ is decreased in EDEM1-transfected HEK293 cells. ELISA analysis of (**A**) Aβ_40_ and (**B**,**C**) Aβ_42_ levels in conditioned media from (**A**,**B**) APP_Swe/Ind_ and (**C**) APP_deltaCT_ cells with or without EDEM1 cotransfection. Values obtained for media of cells without EDEM1 overexpression are indicated as 1. Values representing EDEM1-transfected cells are plotted relative to 1. The values are expressed as mean ± SD, *n* = 3,* *p* ˂ 0.05, ** *p* ˂ 0.01, Student’s *t* test.

We aimed to determine whether reduction in intracellular APP levels induced by EDEM1 overproduction leads to a decrease in Aβ secretion by analyzing the medium obtained from cultures of HEK293 cells with or without overexpression of EDEM1. In cells transfected with the examined APP constructs, EDEM1 overexpression reduced the amounts of secreted Aβ_40_ and Aβ_42_ (Figure 10). We observed an approximately 25% reduction in the level of Aβ_40_ and a 16% reduction in Aβ_42_ in APP_Swe/Ind_ (Figure 10A,B), and an approximately 20% reduction in the amount of Aβ_42_ in APP_deltaCT_ (Figure 10C). These results indicate that a reduction in APP levels in the cell has a significant effect on Aβ forms production. Moreover, this effect is closely related to intracellular Aβ production, as indicated by data obtained for cells transfected with the APP_delta CT_ construct.

## 3. Discussion

Several studies have indicated that the early stages of APP maturation in the ER largely determine the metabolism of this protein. APP can enter different processing pathways, among which the degradation paths significantly affect the fate of APP, including Aβ production. Besides degradation by the ubiquitin-proteasome pathway [39,64,66], APP can be also metabolized in lysosomes [74] and by the stress-responsive chaperone-protease HtrA2 [36]. It has been suggested that the proteasome is involved in the degradation of misfolded forms of APP that lack the correct structure due to blocked N-glycosylation or due to the action of other factors affecting APP maturation in the ER [66]. Before degradation in the cytosol, APP retrotranslocates out of the ER as the ERAD substrate. EDEM1 is one of the main regulators of ERAD involved in the recognition of aberrant proteins in the ER [43,44].

We have demonstrated here that EDEM1 interacts with APP and significantly regulates the intracellular levels of immature forms of APP_695_ and APP_751_, which also affects the amounts of mature forms of APP. Some discrepancies between fold reduction of the immature APP_695_ and APP_751_ in the EDEM1-transfected cells (more than 8-fold reduction for APP_695_ and 4.5-fold reduction for APP_751_) in comparison to immature APP levels in the corresponding control cells may be due to the fact that immature APP_751_ was analyzed together with mature APP_695_ and/or due to possible different interaction partners of APP_695_ and APP_751_ within the ER that alter their regulatory mechanisms. It has been demonstrated that the immature form of APP_695_ interacts with ER proteins to form large complexes [30]. It cannot be excluded that immature APP_695_ and APP_751_ are involved in interactions with different ER proteins. However, a proteomics-based approach did not identify any ER proteins specific to either of these two isoforms [75]. Anyway, it is important that the exact mechanism of APP_695_ and APP_751_ recognition by ER regulatory proteins, especially the ERAD process proteins, is fully understood and future studies should aid in resolving this issue.

The unique effect of EDEM1 on the immature form of APP_695_ was also confirmed in cells with downregulated EDEM1 gene expression, where we observed a dramatic increase in the amount of this form. This effect was observed not only in the non-neuronal kidney cells, but also in human neuroblastoma cells. These results indicate that depletion of EDEM1 can significantly alter the metabolism of endogenously produced APP.

EDEM1-induced reduction in overproduced APP levels was dramatically restored by pretreatment with specific proteasome inhibitors, lactacystin or epoxomycin. These results suggest that a decrease in APP levels by EDEM1 is associated with APP transport to the cytosol and subsequent proteasomal degradation of this protein. Indeed, our permeabilisation assay revealed increased transport of the immature form of APP from the ER to the cytosol in EDEM1-transfected cells. It has been previously reported that immature APP_751_ and the N-terminally truncated form of APP are retrotranslocated to the cytosol [36]. Both of these forms of APP can be degraded by the proteasome and a chaperone protease HtrA2, which is located in the cytosolic side of the ER membrane. The EDEM1-dependent reduction of endogenous levels of APP_695_ and APP_751_ was not completely restored by the proteasome inhibitor, suggesting that probably another degradation system, in addition to the proteasome, is important in these processes. On the other hand, the results of experiments performed in cells with APP overproduction suggest that in cells with elevated APP levels, this protein is mainly degraded by the proteasome.

In our permeabilisation assays, we used a proteasome inhibitor, which allows us to analyze the amount of immature APP_695_ present in the cytosolic fraction. In cells with a functional proteasome, the entire pool of cytosolic APP is degraded very rapidly, as demonstrated previously in [36] and also confirmed in this study. It should be noted that the observed amounts of immature APP present in the cytosol do not reflect the total amount of APP retrotranslocated out of the ER. It was demonstrated that the proteasome activity is required for retrotranslocation for the movement through the retrotranslocation channel, for the ERAD substrate dissociation of the ER protein regulatory complex, or for both steps [76,77]. It is known that proteasome inhibition causes APP retention in the ER under ER stress [64]. Additionally, our mild digitonin treatment released only 20–25% of the cytosolic fraction [59]. A low concentration of digitonin was used to avoid leakage of ER proteins to the cytosol.

It has been reported that the CMV promoter can be significantly upregulated in cells treated with proteasome inhibitors for longer than 8 h, yielding dramatic increases in mRNA and protein levels after 24 h of incubation with proteasome inhibitors [78,79]. In our work, we used EDEM1 cDNA fused to an HA-tag in the pCMV-SPORT2 vector [42]. There were no differences in the cellular EDEM1 levels between cells untreated with proteasome inhibitors and cells treated with proteasome inhibitors for 5 h.

Accumulating observations indicate that ER stress plays an important role in the etiology and pathogenesis of AD [80,81,82]. This was demonstrated in studies using cellular and animal models of this disease, as well as in human postmortem brains. However, the ER-related pathological features of AD appear to be complex and multifaceted. The unfolded protein response (UPR) is triggered in cells subjected to ER stress (for a review see reference [82]). During this response, the chaperone protein BiP/GRP78 is released from the three UPR sensors, leading to their activation. Activation of the first receptor, RNA-dependent protein kinase-like ER kinase (PERK), causes phosphorylation of the translation factor eIF2α, which inhibits protein synthesis. The second effector of this response, transcription factor-6 (ATF6), is responsible for an increase in the amount of ER chaperone proteins, including BiP and calreticulin, which accelerates the folding capacity of the ER. It is known that the immature form of APP_751_ interacts with BiP in the ER [29] and that this interaction is elevated upon ER stress [80]. Under ER stress, BiP retains APP in the ER, prevents its transport to distal compartments, where amyloidogenic proteolysis takes place, which overall directly leads to reduced Aβ production [29,80]. Furthermore, both immature and mature forms of APP_695_ can bind to calreticulin, forming larger complexes that may contain other ER chaperones [30]. However, it should be noted that the nature of calreticulin interaction with immature and mature APP may differ significantly. It has been shown that the mature form of APP can interact with calreticulin to form a complex on the cell surface or alternately reach the cell surface as a complex [31]. Moreover, calreticulin regulates Aβ production as it interacts with sequences at the γ-cleavage site of APP and binds to presenilin and nicastrin to modulate the γ-secretase activity. These observations suggest that overproduction of BiP and calreticulin may promote different mechanisms leading to reduced Aβ generation. This mechanism is even more complex if we consider the action of EDEM1. EDEM1 production during UPR depends on activation of the inositol-requiring protein 1α (IRE1) [49]. IRE1 controls specific splicing of the transcription factor XBP1, which in turn induces the expression of factors facilitating ERAD. This is the third and final stage of the UPR. It can be assumed that during the ER stress, a high level of EDEM1 promotes the transport of the immature form of APP to the cytosol and its further proteasomal degradation. Taking all of these factors into consideration, it can be suggested that the depletion of APP as a substrate in the amyloidogenic pathway during ER stress may be related to its increased interaction with BiP and possibly calreticulin, and to transient retention of APP in the ER [29,80], but also to increased expression of EDEM1, resulting in the final accelerated transport of APP to the cytosol and its increased proteasomal degradation. This is consistent with our experiments performed with proteasome inhibitors and with the observation that APP is preferentially degraded by proteasome during acute ER stress [64]. In a study using a *Drosophila* model of chronic ER proteinopathy in the brain, activation of the IRE1/XBP1 pathway was shown to significantly reduce the Aβ_42_ levels [83]. Moreover, overexpression of either dEDEM1 or dEDEM2 in neurons had decreased Aβ_42_ levels in the brain without significant effects on the Aβ_42_ mRNA levels. XBP1 negatively regulates expression and activity of β-secretase, the rate-limiting enzyme involved in the production of the Aβ peptide, by promoting activity of the ubiquitin-ligase HRD1 [84]. Several studies indicate that XBP1 protects against Aβ toxicity and ameliorates pathology connected with AD [85]. Based on these results, it can be concluded that properly functioning ERAD which attenuates ER stress can lower the toxic Aβ generation in the cell.

However, the capacity and efficiency of ERAD are decreased under pathological conditions caused by chronic ER stress [83]. The continuous accumulation of Aβ and Tau proteins can cause ERAD deregulation and chronic ER stress [83,86]. Furthermore, it was demonstrated that despite activation of the UPR, ERAD activity can be reduced in aged tissues [83]. The mechanisms of UPR signaling and ERAD activation appear to be a complex scenario, and different and even initially conflicting effects have been observed in AD models. It was demonstrated that IRE1 activation in the brain, measured as IRE1 phosphorylation, unexpectedly positively correlates with the severity of AD [81]. In this case, targeting IRE1 signaling has significantly reduced Aβ deposition. It cannot be excluded that altered neuronal proteostasis during AD progression triggers abnormal levels of ER stress and activates the damaging UPR signaling processes mediated in part by the IRE1/XBP1 pathway. Indeed, it was shown that long-term activation of IRE1/XBP1 was detrimental in fly neurons [83]. All of this exacerbates AD. It is possible that under chronic ER stress, the level of EDEM1 is reduced which may result in the abnormal production of APP that is targeted to the amyloidogenic pathway. Furthermore, chronic ER stress inhibits the proteasome [87], which may additionally contribute to the intracellular accumulation of APP and increased production of toxic Aβ.

In conclusion, we found that EDEM1 has a significant effect on regulation of the APP_695_ and APP_751_ metabolism, and production of the Aβ forms. EDEM1 interacts with APP and promotes its retrotranslocation from the ER to the cytosol. This may suggest that this crucial ERAD regulator has an important role in the pathogenesis of AD. Although it was reported previously that overexpression of *Drosophila* EDEMs did not activate canonical UPR (including chronic UPR activation), interestingly it suppressed behavioral deficits and neurodegeneration in Aβ_42_ flies [83]. Thus, selective activation of human EDEM1 may be expected to have a potential therapeutic role in AD.

## 4. Materials and Methods

### 4.1. Reagents and Antibodies

Digitonin was obtained from Merck (Darmstadt, Germany), lactacystin was purchased from Enzo Life Sciences (Farmingdale, NY, USA), and epoxomicin was purchased from UBPBio (Oxfordshire, UK). The mouse monoclonal anti-HA antibodies, anti-rabbit Alexa594 and anti-mouse Alexa488 were obtained from Thermo Fisher Scientific (Waltham, MA, USA). The mouse monoclonal anti-EDEM1, monoclonal anti-β-actin-peroxidase, rabbit anti-APP C-terminal, as well as the secondary anti-rabbit HRP and anti-mouse HRP antibodies were from Merck (Darmstadt, Germany). The mouse monoclonal anti-APP were obtained from OriGene (Rockville, MD, USA), the rabbit anti-PDI were from Cell Signaling (Danvers, MA, USA). The mouse anti-calreticulin was from Enzo Life Sciences (Farmingdale, NY, USA), whereas mouse monoclonal anti-calnexin was from BD Biosciences (Bedford, MA, USA). The mouse anti-HSP70 was obtained from Santa Cruz Biotechnology (Dallas, TX, USA).

### 4.2. DNA Constructs

The mouse EDEM1 cDNA fused to an HA-tag in the pCMV-SPORT2 vector was a kind gift obtained from Prof. Kazuhiro Nagata and Prof. Nobuko Hosokawa (Institute of Frontier Medical Science, Kyoto University, Kyoto, Japan) [42]. All APP constructs came from the laboratory of Prof. Dennis Selkoe and Dr. Tracy Young-Pearse (Brigham and Women’s Hospital, Harvard Medical School, Boston, MA, USA): pCAX APP 695 (Addgene plasmid #30137; Available online: http://n2t.net/addgene:30137 accessed on 26 September 2016; RRID:Addgene_30137), pCAX APP 751 (Addgene plasmid #30138; Available online: http://n2t.net/addgene:30138 accessed on 26 September 2016; RRID:Addgene_30138), pCAX APP Swe/Ind (Addgene plasmid # 30145; Available online: http://n2t.net/addgene:30145 accessed on 15 November 2018; RRID:Addgene_30145), pCAX APP delta CT (Addgene plasmid # 30143; Available online: http://n2t.net/addgene:30143 accessed on 15 November 2018; RRID:Addgene_30143) [71]. The esiRNAs (endoribonuclease-prepared siRNAs) against EDEM1 and GFP (used as a negative control) were obtained from Merck. Alternatively, for EDEM1 downregulation small interfering vector-based RNAs (shRNAs) against EDEM1 (shEDEM1) were applied [59].

### 4.3. Cell Culture and Transfections

Human Embryonic Kidney 293 (HEK293; Merck, Darmstadt, Germany) and human neuroblastoma (SH-SY5Y) cells were maintained in Dulbecco’s Modified Eagle Medium (DMEM; Corning, NY, USA) supplemented with 10% fetal bovine serum (FBS; Eurx, Gdańsk, Polska), 25 U/mL penicillin, and 25 µg/mL streptomycin (Merck, Darmstadt, Germany) in a humidified incubator at 37 °C and 5% CO_2_. Cells were transiently transfected with 2 µg/well (6 well-plates) or 4 µg/plate (6 cm plate) cDNA using TurboFect transfection reagent (Thermo Fisher Scientific, Waltham, MA, USA), according to the manufacturer’s procedure. For cotransfection with two different cDNAs, half the amount of cDNA was used. In analyses in which APP/EDEM1-transfected cell samples were compared with APP-transfected cells, the latter were cotransfected with the appropriate APP cDNA and control cDNA. For the esiRNA transfection, 40 nM esiEDEM1 was applied with ScreenFect transfection reagent (InCella, Eggenstein-Leopoldshafen, Germany), according to manufacturer’s procedure.

### 4.4. Total RNA Isolation and Quantitative Real-Time RT-PCR

Total RNA was isolated from HEK293 or SH-SY5Y cells transfected with esiEDEM1 or shEDEM1 and from HEK293 cell transfected with EDEM1-HA by RNA Extracol (Eurx, Gdańsk, Polska), following the protocol provided by the manufacturer. Total RNA was then treated with 2 units/per 10 µg RNA of TURBO DNase (RNase-Free, 2 U/μL, Thermo Fisher Scientific, Waltham, MA, USA) at 37 °C for 30 min, according to the manufacturer’s procedure. The TURBO DNase was inactivated at 75 °C for 10 min. The overall quality of RNA preparations was assessed by electrophoresis on a denaturing agarose gel. RNA was quantified using Quant-it RiboGreen RNA Assay Kit (Thermo Fisher Scientific, Waltham, MA, USA) according to the protocol provided by the manufacturer. RNA integrity, purity, concentration, and size were also analyzed by the Agilent 2100 Bioanalyzer (Agilent, Palo Alto, CA, USA) using the RNA 6000 Nano assay (Agilent), according to the manufacturer’s instructions and using the Agilent 2100 Bioanalyzer Software (Agilent, Palo Alto, CA, USA). The highest quality RNA, with visibly distinct 18S and 28S ribosomal RNA bands, not degraded and with a RNA Integrity Number (RIN) above 8.0 was used for real-time qRT-PCR. Then, 500 ng of total RNA were reverse-transcribed into cDNA using the Transcriptor First Strand cDNA Sythesis Kit (Roche Life Science, Basel, Switzerland) according to the manufacturer’s instructions.

The real-time PCR reactions were performed using the Roche LightCycler TaqMan Master mix in combination with Roche Universal Probe Library (UPL) assays. All assays were designed to span an intron-exon boundary to prevent amplification of DNA. Each reaction consisted of 1 × LightCycler TaqMan master mix, specific primer pairs and fluorescently-tagged probes, both for the reference and the examined gene. The expression of glyceraldehyde-3-phosphate dehydrogenase (GAPDH) mRNA was used as reference control. The primers and fluorescently-tagged probe for the reference gene were used according to the protocol provided by the manufacturer (Roche Life Science, Basel, Switzerland). It has been evaluated previously that down-regulation of EDEM1 or overexpression of EDEM1-HA does not influence the expression of the reference gene [60]. The real-time PCR reaction was performed in the LightCycler 480 System (Roche Diagnostic, Basel, Switzerland) under the following conditions: initial denaturation, incubation at 95 °C for 10 m followed by: 45 cycles of 95 °C denaturation for 10 s, 60 °C annealing for 30 s, and extension at 72 °C for 1 s. Quantification of mRNA expression was carried out using the LightCycler detection system. Real time PCR efficiency was calculated for each reaction, and only results from reactions whose efficiencies were within the range of 90–110% (corresponding to a 1.8–2.2-fold increase per cycle) were exclusively taken for further analysis. The efficiency correction was accounted for in mRNA quantification. The 2^−ΔΔct^ method was used to determine the relative gene transcript levels after normalization to the reference genes [88].

### 4.5. Cell Lysis and Western Blotting

HEK293 or SH-SY5Y cells were seeded in 6 cm plates (1 × 10^6^/plate) or 6-well plates (5 × 10^5^/well) and transfected with the appropriate cDNAs or esiEDEM1. Then 72 h post transfection, cells were washed with ice cold phosphate-buffered saline (PBS) and lysed in a buffer (0.1 M NaCl, 10 mM Na_2_HPO_4_, 1 mM EDTA, 1% Triton X-100, pH 7.4) supplemented with protease inhibitor mixture (Roche Life Sciences, Basel, Switzerland). Alternatively, before lysis the cells were incubated for 5 h at 37 °C with epoxomicin (1 µM) or lactacystin (10 µm). Lysates were centrifuged to remove cell debris and nuclei for 10 min at 10,000× *g*. Samples were resolved by reducing SDS/PAGE (12% gels). The proteins were transferred onto an Immobilon-FL membrane (Merck, Darmstadt, Germany) by the Trans-Blot Turbo Transfer System (Bio-Rad, Hercules, CA, USA). Membranes were then incubated with appropriate primary and secondary antibodies. Proteins were detected by chemiluminescence with the Clarity Max ECL Western Blotting Substrate (Bio-Rad, Hercules, CA, USA) or the SuperSignal West Femto Maximum Sensitivity Substrate (Thermo Fisher Scientific, Waltham, MA, USA) and were visualized using Typhoon FLA 9400 (GE Healthcare, Chicago, IL, USA). Signal intensities of the bands were quantified using Image Studio Lite (v.5.2) (LI-COR Bioscences, Lincoln, NE, USA).

### 4.6. Retrotranslocation Assay

HEK293 cells were seeded in 6 cm plates (1 × 10^6^/plate) and cotransfected with APP_695_ and EDEM1-HA cDNAs or APP_695_ and control cDNAs. Then 72 h post transfection, the cells were incubated for 5 h at 37 °C with epoxomicin (1 µM) and washed with room temperature PBS. For permeabilisation, the cells were incubated for 5 min at room temperature with KOAc buffer (115 mM CH3COOK, 25 mM HEPES, and 2.5 mM MgCl_2_, pH 7.4) containing 3 μg/mL digitonin, followed by a 30-min incubation on ice. After incubation, the buffer containing cytosolic fraction was collected, and the remaining membranes were lysed as indicated above. Both the cytosolic and membrane fractions were centrifuged for 10 min at 10,000× *g*. APP was immunoprecipitated from both fractions with mouse anti-APP antibodies immobilized on Dynabeads protein G (Thermo Fisher Scientific). Finally, the beads were washed with ice-cold PBS supplemented with 0.35% Triton X-100, and the adsorbed material was resolved by SDS/PAGE (12% gels) under reducing conditions. For the detection of APP by Western blotting, the proteins were transferred onto an Immun-Blot Low Fluorescence PVDF membrane (Bio-Rad, Hercules, CA, USA). In addition, Western blotting was performed for the cytosolic and membrane fractions with anti-calreticulin, anti-calexin, and anti-Hsp70 antibodies and the corresponding secondary antibodies.

### 4.7. Co-Immunoprecipitation Assay

HEK293 cells were seeded in 6 cm plates (1 × 10^6^/plate) and cotransfected with APP_695_ and EDEM1-HA, APP_695_ and control, EDEM1-HA and control, or control cDNAs. Then 72 h post transfection, the cells were washed with cold PBS and lysed as indicated above. Samples were then sonicated (5 min, 40% output). The lysates were centrifuged to remove cell debris and nuclei at 10,000× *g*. The supernatants were immunoprecipitated for 1 h at 4 °C using mouse anti-APP antibodies coupled to protein G Dynabeads (Thermo Fisher Scientific, Waltham, MA, USA). The beads were washed 3 times with HBS buffer (pH 6.8) containing 0.1% Tween 20. Samples were resolved by 12% reducing SDS/PAGE and transferred onto a PVDF membrane (Merck, Darmstadt, Germany). For the Western blotting detection of EDEM1 interacting with APP, membranes were treated with anti-HA antibodies. For the control of APP_695_ and EDEM1 transfection, whole cell lysates were subjected to Western blotting with anti-APP or anti-HA antibodies.

### 4.8. Immunofluorescence Microscopy

HEK293 cells cotransfected with APP_695_ and EDEM1-HA or cells transfected with EDEM1-HA were grown on coverslips. Cells were then washed once with PBS and fixed in 2% (*w/v*) paraformaldehyde (PFA, Merck, Darmstadt, Germany). Cells were then permeabilised in 0.1% Triton X-100 and blocked in 5% FBS before labelling with mouse anti-HA together with rabbit anti-APP or with mouse anti-HA together with rabbit anti-PDI and treated with appropriate secondary antibodies. DAPI (Thermo Fisher Scientific, Waltham, MA, USA)) was used to stain the nuclei. The cells were mounted in Mowiol (Molecular Probes, Eugene, OR, USA) and examined with a laser scanning confocal microscope LSM800 Zeiss with Airyscan (Carl Zeiss, Jena, Germany) or with an automated inverted microscope Leica DMI4000B (Leica, Wetzlar, Germany). Images were prepared with the ZEISS ZEN Microscope Software (Carl Zeiss) or the Leica Application Suite 3.1.0 (Leica) and were analyzed by the Fiji plugin in the ImageJ software. Mander’s coefficient was used for reporting co-localization between EDEM1 and APP, APP and EDEM1, or EDEM1 and PDI. Mander’s coefficient ranges from 0 to 1, corresponding to non-overlapping images and 100% co-localization between the two images, respectively.

### 4.9. Analysis of APP and Aβ Forms by ELISA

APP, Aβ_40,_ and Aβ_42_ levels were quantified using commercially available, standard ELISA kits (Thermo Fisher Scientific, Waltham, MA, USA) according to the manufacturer’s protocol. For APP detection, HEK293 lysates of cells transfected with EDEM1-HA or control cDNAs, or cotransfected with APP_695_ and EDEM1-HA or APP_695_ and control were used. For Aβ detection, conditioned media coming from cells cotransfected with APP_695Swe/Ind_ and EDEM1-HA or APP_695Swe/Ind_ and control cDNAs, as well as from cells cotransfected with APP_deltaCT_ and EDEM1-HA or APP_deltaCT_ and control cDNAs were applied. To prevent degradation of APP or Aβ, protease inhibitors (Roche Life Sciences, Basel, Switzerland) were added. The lysates or the conditioned media were loaded onto plates coated with an appropriate APP or Aβ-specific antibodies and treated with corresponding detector antibodies. ELISA plates were developed using a color reaction, and the absorbance was read at 450 nm using the Victor 3 plate reader (Perkin Elmer, Waltham, MA, USA).

### 4.10. Statistics

All experiments were performed independently at least three times. Values are expressed as mean ± SD. Statistical analyses were performed by Student’s *t* test. A *p* value of 0.05 or less was considered to be statistically significant. *p*-values of * *p* ˂ 0.05, ** *p* ˂ 0.01, *** *p* ˂ 0.001.

## Data Availability

Data and tools described in this manuscript are available upon request.

## Supplementary Materials

The following are available online at https://www.mdpi.com/article/10.3390/ijms23010117/s1.
