# Supplementary material for: EDEM1 Regulates Amyloid Precursor Protein (APP) Metabolism and Amyloid-β Production
Source: Int J Mol Sci. 2021 Dec 23;23(1):117. doi: 10.3390/ijms23010117 (PMC8745108; doi:10.3390/ijms23010117)
Supplement: Supplementary file 1 [file ijms-23-00117-s001.zip › ijms-1484508-supplementary.pdf]

Supplemantary Materials

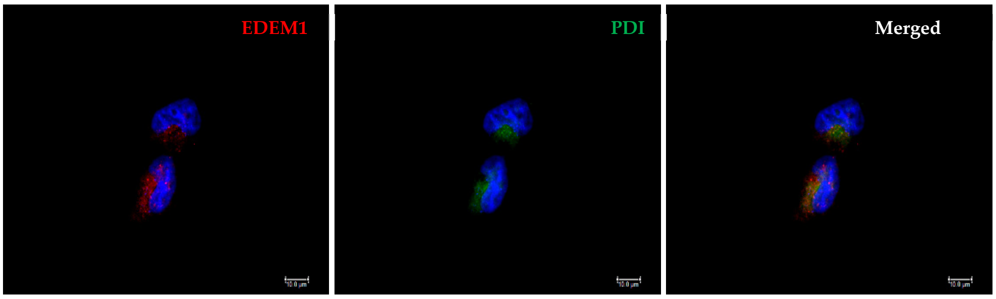

Figure S1. EDEM1 colocalizes with PDI in HEK293 cells. Cells were transfected with EDEM1, and were then fixed and stained as indicated. DAPI was used to stain the nuclei. Bars, 10μm. Control staining without anti-EDEM1 or anti-PDI antibodies confirmed specific recognition by secondary antibodies.
